# Potential factors that can affect the performance of undergraduate pharmacy research students: a descriptive study

**DOI:** 10.1186/s12909-023-04018-5

**Published:** 2023-01-17

**Authors:** Danijela Gnjidic, Narelle da Costa, Nial J. Wheate

**Affiliations:** Sydney Pharmacy School, Faculty of Medicine and Health, The University of Sydney , NSW 2006 Sydney, Australia

**Keywords:** Undergraduate, Research, Pharmacy, Supervision, Performance, Honours

## Abstract

**Objective:**

This descriptive study aimed to examine whether student past coursework performance, student or research supervisor characteristics, and the type of research project are related to the overall academic performance of a pharmacy student completing an honours research program.

**Methods:**

Data on undergraduate honours students who completed a Bachelor of Pharmacy degree at The University of Sydney, Sydney, Australia, between Jan 2015 and Dec 2020 was collected. This included socio-demographic characteristics, type of project undertaken, and academic outputs. Data was also collected on each supervisor’s academic role, level of experience, research area, and where they completed their PhD. Descriptive statistics were used to describe the study cohort and correlation analysis and unpaired t-tail analyses were conducted using SPSS software.

**Results:**

This five year study included 130 students of which 67% were female and 60% were domestic students. Each student was supervised by one of 48 individual academics who were a mix of early- (31%), mid-career (29%), and experienced researchers (40%) for pharmaceutical science (50%), clinical (45%), and education (5%) projects. Just less than half (49%) of students published one peer-reviewed journal article. Female students outperformed male students (*p* = 0.031) with female students also twice as likely (15%) to receive a university medal eligible mark compared with male students (7.0%). Similarly, domestic students were twice as likely (15%) to receive a university medal eligible mark when compared with international students (7.7%). Students who undertook a pharmaceutical science-based project outperformed education-based project students (*p* = 0.0235). Students who had published at least one peer-reviewed journal article outperformed those who had not published (*p* = 0.0014).

**Conclusion:**

Factors that affected honours performance were student gender, residential status, type of project undertaken, and whether a student had published a peer-reviewed journal article.

## Introduction

Undergraduate student performance varies across individual degrees, year cohorts, and disciplines. A major factor in how well an individual student performs is related to their natural ability, but comprehensive research at the high school and university levels has demonstrated a myriad of other factors that can also affect performance [1]. This includes student personal characteristics, their home environment, and the teaching environment.

When it comes to the student themselves, research has shown that the goals they set, and their level of anxiety, may affect their academic performance [2]. Students who attend classes regularly [3] and students who undertake personal reflection on what they have learnt tend to do better [4]. Even a student’s gender is a factor, with one study showing that female students across disciplines tended to have better performance when compared with male students [5]. An additional important factor that has been demonstrated is the student’s comprehension of English, where classes are taught in English [6].

When comparing across educational environments, factors such as the level and type of communication (written and oral) with students, what learning facilities and resources are available to students, and the guidance they receive from academics [6] can affect their research performance. Students tend to perform better when poor performance is identified early, and extra support is given [7].

To date, most research that has been undertaken on the factors that affect undergraduate student performance has focussed entirely on their outcomes for coursework learning. There are no studies that examine how different factors affect a student’s performance in undertaking formal research at the undergraduate level (e.g. honours).

In the Australian higher education system, undergraduate students can elect to undertake a formal research component of their degree called honours. Depending on the degree being studied, honours can comprise specific courses/units of study (UoS) as part of a bachelor’s program (referred to as an embedded or integrated honours), or it may comprise a separate appended year at the completion of a bachelor’s degree.

Across the world, at universities that provide bachelor degrees that follow a typical British structure, for example England, New Zealand and Australia, students have the opportunity to undertake honours research as part of their degree. Degrees provided in this structure generally follow a three- or four-year course of taught classes and a formal research component that students enrol into and for which a grade/mark is awarded. Currently, pharmacy students at The University of Sydney, Sydney, Australia are able to undertake embedded honours research in the 4th year of the Bachelor of Pharmacy degree or the 5th year of the Bachelor of Pharmacy and Management degree.

Given the individual nature of honours, where a student works one-on-one with a supervision team (lead by a designated primary supervising academic), there can be additional factors not related to natural ability that can potentially affect their performance. As such, it is not surprising that there is variability in student outcomes, but it is unclear what factors most influence performance.

It was therefore of interest to examine what factors can impact performance in the current pharmacy honours program. In this descriptive study, we aimed to examine whether past performance, student or supervisor characteristics, and the type of project undertaken could influence the overall performance of a student completing undergraduate honours research projects.

## Methods

### Ethics

This study was approved by The University of Sydney Human Ethics Research Committee (No. 2021/44).

### Data collection

Data for all students who completed honours between January 2015 and December 2020 as part of the Bachelor of Pharmacy (BPharm) or Bachelor of Pharmacy and Management (BPharm & Management) degree programs through the School of Pharmacy at The University of Sydney was obtained for the study. This included each student’s gender, residency status (domestic or international), their weighted average mark (WAM; defined as the average mark a student achieved across all completed units in their degree course) on entry into honours, whether they were a named author on a published peer-reviewed journal article and their number of authored articles, their marks in the two UoS that comprise the honours program (PHAR4815 – Research Methods and PHAR4830 – Honours), and their final overall honours grade (Honours Class 1, Class 2 Division 1, or Class 2 Division 2). Data was also collected on each student’s primary academic supervisor with regard to their level of experience, where they completed their PhD (Australia or overseas), their academic role (teaching and research (T&R) academic, education-focussed academic, or research-focussed academic), and their broad research area (pharmaceutical science, clinical, or education).

A student’s project was classified based on the broad research area of their primary supervisor. A pharmaceutical science project was defined as laboratory-based research in medicinal chemistry, pharmaceutical chemistry, or biology. Education projects were defined as those that included research into pharmacy teaching and learning, and clinical projects were defined as research into social pharmacy, pharmacy practice and the quality use of medicine, or hospital-based projects. Between 2015 and 2020 pharmacology research was not undertaken in the School of Pharmacy, but was a part of the School of Medical Sciences, and therefore no pharmacology projects were included in this research study.

Each supervisor’s academic experience was classified into one of three categories: early career academics (ECR) who were defined as having completed their PhD between the calendar years 2011 and 2020, mid-career researchers (MR) who were defined as having completed their PhD between 2001 and 2010, and established researchers (ER) who were defined as having completed their PhD in the year 2000 or earlier.

### Student assessment and grading

The honours program consisted of two units of study: PHAR4815 – Research Methods and PHAR4830 – Honours. PHAR4815 is a six credit point unit delivered in semester 1 of each year. While completing PHAR4815, the honours students also complete three other six credit point UoS; PHAR4811 - Pharmacotherapeutics, PHAR4812 – Integrated Dispensing and Practice, and PHAR4823 – Pharmacy Services and Public Health, which do not contribute to their honours mark. As part of PHAR4815, students learnt basic research skills such as referencing, statistics, and data management. Students also research the background to their assigned project and prepared a literature review. The actual research project is conducted as part of the PHAR4830 UoS in semester 2 and comprises the entire semester workload (24 credit points).

**Fig. 1:**
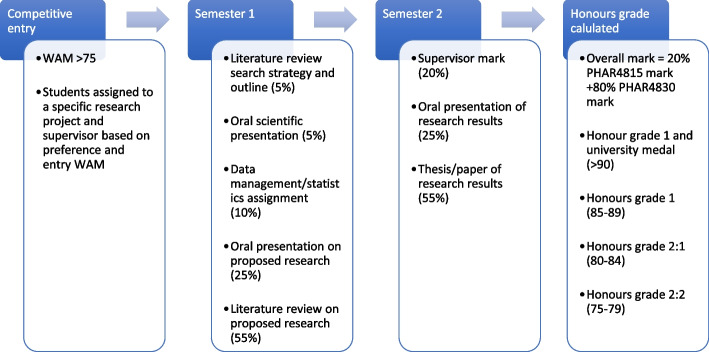
A flow chart of the pharmacy honours program showing how students are assessed (assessment weighting) and how final overall honours grades are calculated

Different assessment methods were used to grade the students on their honours performance and to decide their final honours level (Fig. 1). The major assessments for PHAR4815 and PHAR4830 are in the forms of a research paper. The literature review comprises either a narrative or systematic review depending on the nature of the project and the preference of the supervisor. The research thesis is prepared as a draft journal article; there have been instances where students have published their research results in a peer-reviewed journal before the thesis due date. In those instances, the students are permitted to submit the accepted/published journal article as their thesis.

On completion of honours, students receive one of three honours grades: Class 1, awarded for an overall honours mark of 85 or higher, Class 2 Division 1 (Class 2:1), awarded for an overall honours mark of 80–84, and Class 2 Division 2 (Class 2:2), awarded for an overall honours mark of 75–79. A student who achieves a honours mark lower than 75 would not generally be awarded honours but instead given a pass degree. In addition to the three honours classes, students could also be awarded a university medal. The award of the medal is at the discretion of the faculty, with the minimum requirements of an overall degree WAM of 85 and an overall honours mark of at least 90. The university medal is the highest undergraduate award given by The University of Sydney.

### Student supervision

Each honours research student has a formally appointed primary academic supervisor who is responsible for the design of the student’s research project and provides mentoring to the student. The primary academic supervisor is often supported by a secondary academic supervisor (also called a co-supervisor or auxiliary supervisor) who may have varying levels of engagement in student mentoring and the project. The academic supervisors were of any one of five academic levels (Level A, associate lecturer; level B, lecturer; level C, senior lecturer; level D, associate professor; and level E, professor).A staff member could not be a primary supervisor to a student until they had experience as a secondary supervisor.

### Data analysis

Student data was matched to supervisor data and deidentified by one member of the research team (N.dC.) prior to data analysis by the other research team members (DG and NW). Descriptive statistics were performed to analyse the students using proportions and mean (range). In academic research, the most objective marker of research quality is its publication in a published peer-reviewed journal. It was therefore of interest to examine whether there was a correlation between a student named as an author of a journal article and their overall honours performance. For the purposes of this study, a journal article was included in the data set if the student was named on an article that was published before, during, or after their honours and regardless of whether they were first, middle, or last author. Correlation analysis and unpaired t-tests to calculate p scores were undertaken using SPSS 1.0.0.1327 (IBM, New York).

## Results

Over the five year study period, 130 students completed honours, with the student and supervisor characteristics given in Table 1. The percentage of female students was 67% and the percentage of domestic students was 60%. Of the 130 honours students who entered the program, 128 (98%) had a WAM of 75 or greater. Two students who were admitted to the program had WAMs slightly lower than the normal entry requirement (WAMs: 72.8 and 73.3).

**Table 1:** Student and supervisor demographic characteristics

**Student characteristics**	
Gender	87 (67%) female, 43 (33%) male
Residential status	78 (60%) domestic, 52 (40%) international
Average WAM on entry	82.04 (min: 72.8; max: 95.0)
Number of students who published a journal article	64 (49%)
Performance in PHAR4815	Average 84.07 (min: 73; max: 90)
Performance in PHAR4830	Average 86.30 (min: 72; max: 95)
Overall honours performance	95 (73%) Class 1, 29 (22%) Class 2:1, 6 (5%) Class 2:2
**Supervisor characteristics**	
Academic level	18 (31%) ECR, 17 (29%) MCR, 23 (40%) ER
Area of specialisation	29 (50%) Pharm Science, 26 (45%) Clinical, 3 (5%) Education
PhD location	38 (66%) Australia, 19 (33%) international, 1 (1%) No PhD
Academic role	50 (86%) T&R, 6 (10%) Education focussed, 2 (4%) Research focussed

**Fig. 2:**
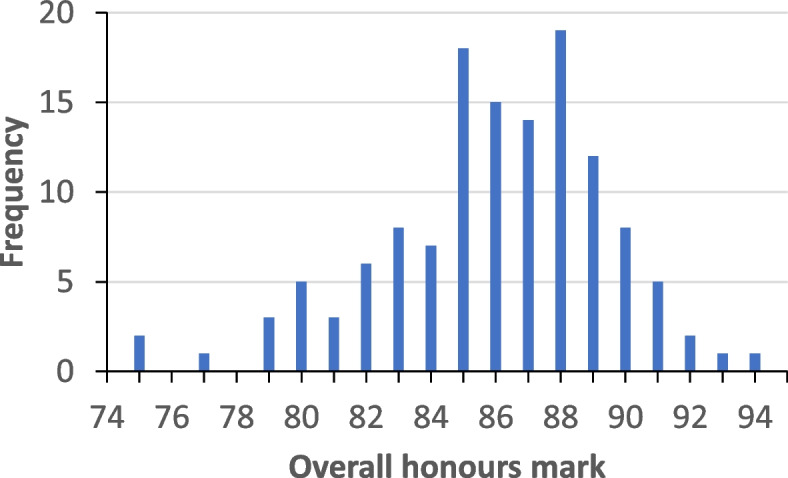
A histogram of the frequency of overall honours marks; a mark of 90 and above is Honours Class 1 and eligible for university medal, a mark of 85–89 is Honours Class 1, a mark of 80–84 is Honours Class 2 Division 1, and a mark of 75–79 is Honours Class 2 Division 2

The average overall honours mark was 86 with a standard deviation of 3 and was normally distributed (Fig. 2). Sixteen (12%) students received an overall honours mark that would have made them eligible for a university medal.

**Fig. 3:**
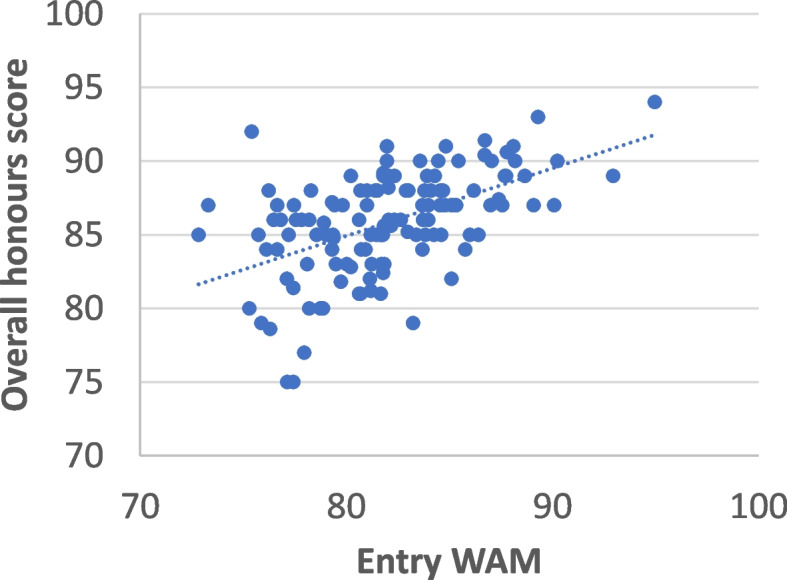
Correlation between student’s weighted average mark (WAM) on entry to honours against their overall performance in honours

When the overall honours marks of the students were compared with their WAM on entry into the program, there was a weak correlation between the two (Fig. 3; correlation analysis R^2^ = 0.28). For those students who entered the degree with an average grade of distinction (mark of 75–84), 70 students (68%) went on to achieve Class I honours. This was considerably lower than the percentage of students who entered the program with an average grade of high distinction (mark of 85 or higher) of whom 25 (93%) achieved first class honours. Two students entered the program with only an average of a credit grade (mark of 64–74) both of whom achieved Class 1 honours. For the students who entered with a distinction average the lowest and highest overall marks achieved by this cohort were 75 and 92, respectively. For the students who entered with a high distinction average, their lowest and highest overall honours marks were 82 and 94, respectively.

**Fig. 4:**
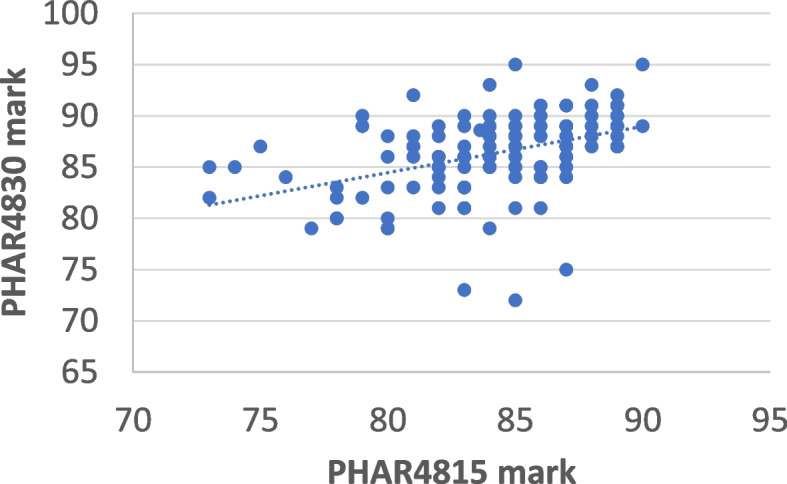
Correlation between student’s performance in semester 1 (PHAR4815; research coursework) against their performance in semester 2 (PHAR4830; research project)

The correlation between the student’s performance in the first honours unit (PHAR4815) was compared with their performance in the second honours unit (PHAR4830) with the results indicating again weak correlation when compared with their marks on entry into the program. Correlation analysis of 4815 to 4830 resulted in an R^2^-value of 0.18 (Fig. 4). Of the students who only achieved a distinction grade for PHAR4815, 43 (66%) went on to achieve Class 1 honours. In comparison, of the 65 students who achieved a high distinction grade for PHAR4815, 54 (87%) achieved a final grade of Class 1. Of the three students who achieved only a credit grade for PHAR4815, two (66%) went on to be awarded Class 1 honours.

No statistically significant difference (*p* = 0.2531) was observed between domestic and international students in overall research performance. The 78 domestic students in the cohort had an average mark of 86.1 compared with an average mark of 85.4 for the 52 international students. Fifty-two of the domestic students (74%) achieved Class 1 honours, compared with the international cohort whose Class 1 honours success rate was 69%. The number of students who achieved an overall honours mark (90 or higher) that would make them eligible for a university medal was higher for domestic students 12 (15%) compared to the international students; four students (7.7%).

**Fig. 5:**
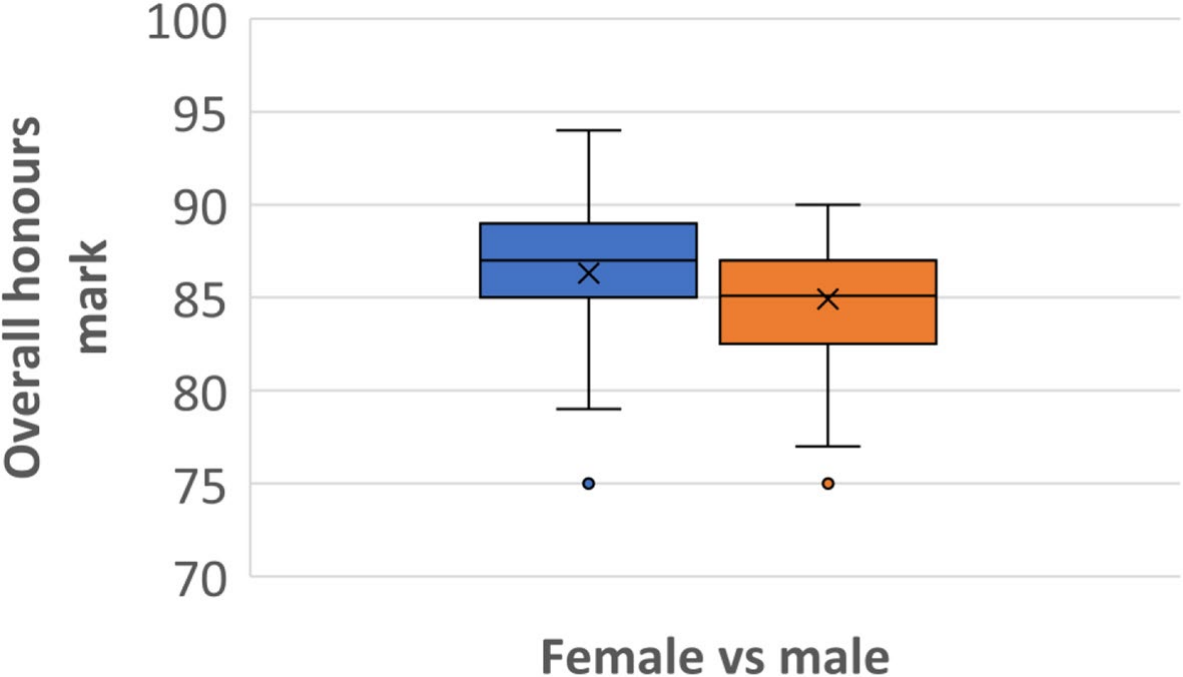
Comparison of overall honours marks achieved by female (blue, left) and male (orange, right) students showing the mean marks (x) and the mark distributions for each gender

There was a statistically significant difference in overall performance according to gender (*p* = 0.031) although the magnitude of the difference in performance between the two groups was small. The average overall honours mark for women was 86.3 compared with just 84.9 for men (Fig. 5). As well as achieving a higher average mark, female students were more likely to achieve Class 1 honours (success rate 76%) compared with male students (65%), and women (13 students of 87 in the sample) were more likely to achieve a university medal eligible mark (15%) compared with men (3 of 43 students, 7.0%).

There was no statistical difference between students who had a supervisor trained in Australia in research compared with a supervisor trained overseas (*p* = 0.930). The Australian-trained supervisor cohort had an average overall honours mark of 85.9 compared with an average mark of 85.8 for the international cohort. Of the Australian-trained supervisors, 70 (76%) of their students achieved honours Class 1, compared with 25 (66%) of the students with overseas trained supervisors.

There was no difference in student academic performance across projects supervised by early career (ECR) academics when compared with mid-career (MR) academics, and established (ER) academics (ECR vs. MR, *p* = 0.7561; ECR vs. ER, *p* = 0.7232; MR vs. ER, *p* = 0.9249). Students who had an ECR supervisor had an average mark of 86.0, compared with 85.8 for MR supervised students, and 85.7 for ER supervised students. A student who was supervised by an ECR academic had a Class 1 honours success rate of 73%, which was not much different to either the MR (70%) or ER (74%) supervised students.

The vast majority of students in this study had primary supervisors who were a T&R academic (119, 92.5%), with only two students (1.5%) supervised by a research-focussed academic, and nine (7%) students by an education-focussed academic. There was no statistical difference in student marks between the three groups. The average mark for the T&R supervised students was 86.0, for education-focussed supervised students it was 83.8, and for research-focussed supervised students it was 85.5. The p score of T&R students against education students was 0.0636. Both of the students who were supervised by a research-focussed supervisor gained Class 1 honours (100%) compared with only three students (33%) of the education-focussed supervisors, and 87 (73%) of the T&R supervised students. Importantly, all students who received a university medal eligible mark (16 students) were supervised by a T&R academic.

**Fig. 6:**
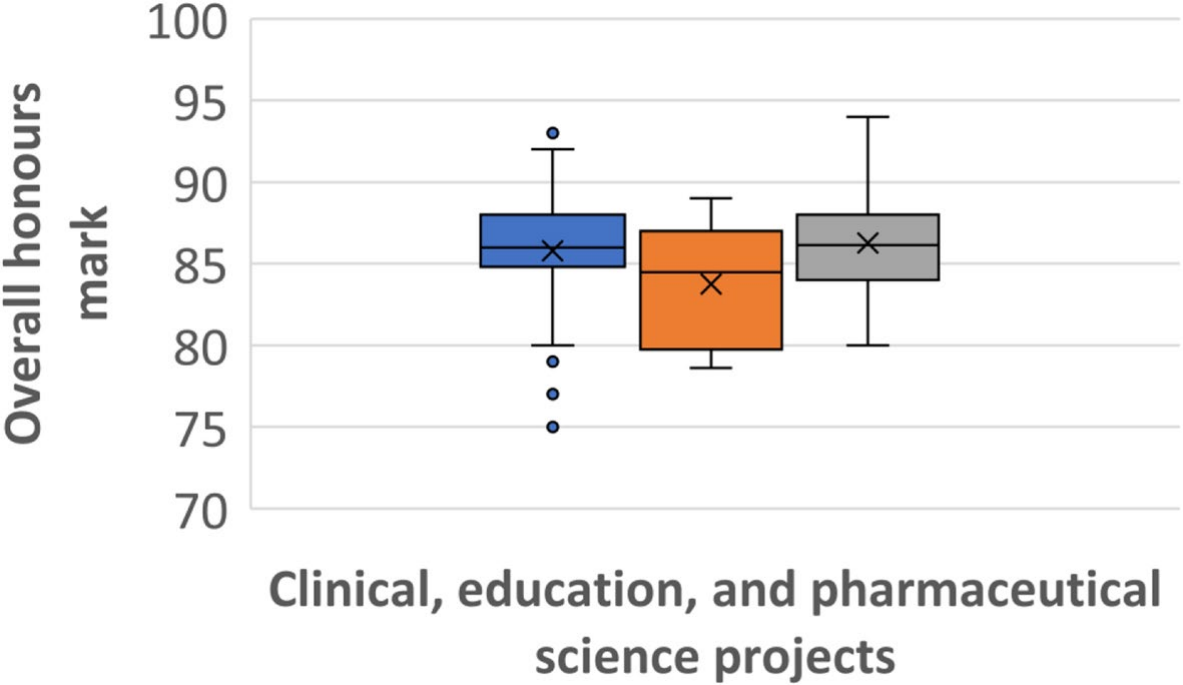
Comparison of overall honours marks achieved by students undertaking clinical (blue, left), education (orange, middle), or pharmaceutical science (grey, right) projects, showing the mean marks (x) and the mark distributions for each group

Clinical (62 students, 48%) and pharmaceutical science (59, 45%) projects were undertaken by the majority of students with only 9 (7%) students completing an education-based project. There was a statistical difference in the overall performance of students who undertook education-based projects (average mark 83.8) and those who completed a pharmaceutical science (average mark 86.3) project (*p* = 0.0235; Fig. 6). There was no statistical difference in the overall mark of education- and clinical-based project (average mark 85.8) students with a p score of 0.4465, and no difference between the pharmaceutical science and clinical project students (*p* = 0.1453). No student who completed an education-based project received a university medal eligible mark while students at this level were almost evenly distributed between clinical (9 students, 14.5%) and pharmaceutical science (12%).

**Fig. 7:**
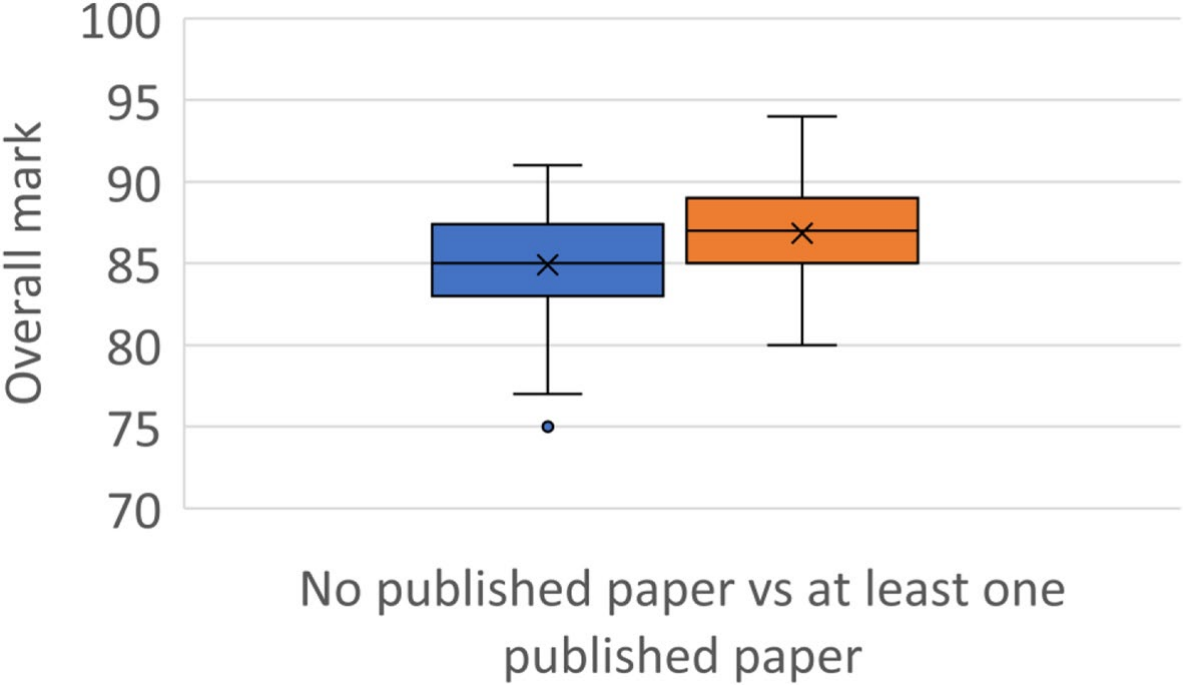
Comparison of overall honours marks achieved by students who had not (blue, left), or had (orange, right), been named as an author on any published peer-reviewed journal article, showing the mean marks (x) and the mark distributions for each group

Of the honours cohort, 64 (49%) were listed as an author on a published peer-reviewed journal article. Thirty-four students had published one journal article, 15 students had 2 journal articles, 10 students had 3 articles, 2 students had 4 articles, another 2 students had 5 journal articles, and a single student had 6 journal articles. There was a statistical difference (*p* = 0.0014) in the overall marks of the students with an average mark of 86.8 for those students named as an author on at least one published article, and an average mark of 84.9 for those students who had not authored a published article (Fig. 7). Students named as an author on a published journal article (11 students, 17%) were more than twice as likely to receive a university medal eligible mark when compared with unpublished students (7.6%).

Of the 64 students who were named as an author on a journal article, 26 (41%) students were supervised by an ECR academic, 23 (36%) by a MR academic, and 15 (23%) by an ER academic. More students who undertook a clinical project (38, 62% of all clinical students) published a paper, compared with pharmaceutical science project students (25, 42%) and education project students (1, 11%).

## Discussion

To our knowledge, this is the first study to investigate which characteristics may predict pharmacy honours student performance. Overall, our findings indicate that student gender, residency status (international or domestic student), and project type can influence student performance. The findings also suggest that students who publish a peer-reviewed journal article are more likely to perform better during their honours year.

Our study suggests that female students outperformed male students, and while the domestic and international student cohorts both had statistically similar average overall honours marks, domestic students were more successful at achieving university eligible marks when compared with international students. The better performance of women over men appears consistent with other studies in gender performance in higher education. Studies consistently show that women outperform men, particularly in science, technology, engineering, and mathematics [5]. In a recent article that examined gender in life and physical science study, women obtained statistically higher course scores compared with men, and were 1.5 times more likely to obtain top grades [8]. Interestingly, men outperform women when assessments are based on high-stakes, multiple-choice, and time limited exams. When assessments are more based on constructed-response exercises, as is the case when undertaking research and preparing a thesis, women outperform men [9].

Moreover, the difference in performance of very high performing domestic vs. international students may be due to English language skills. Given that Class I honours criteria requires high level written language skills and critical evaluation of the current evidence, it is possible that poorer written English skills may be a factor in international students having a lower rate of university medal eligible marks compared with domestic students. This is consistent with more broad education research which has shown that the English proficiency of students on entry to university is related to their overall academic performance [10].

The type of project undertaken by the student was seen to have a potential effect on the overall performance. Specifically, there was a difference in the performance between the pharmaceutical science and the education project students with students undertaking education-based project less likely to achieve higher honours grades. This observation may be because the majority of academic staff with expertise in pharmaceutical or clinical research did not have the expertise to adequately evaluate the education-based research. Alternatively, the extra teaching workload of an education-focussed academic may mean they have less experience in undertaking education research, and so, have difficulty in provided mentoring to students undertaking those types of projects. This finding warrants future investigation.

The study also demonstrates that students performed better if they had published a journal article. At most universities, including The University of Sydney, high performing undergraduate students may be given the opportunity to participate in summer research projects. Having prior research experience through something like a summer research project, even if it is not related to their specific honours project, is likely to provide an advantage to those students. The association between having a published article and being awarded a university medal eligible mark is potentially related not just to the quality of the research undertaken by the student but also their ability to prepare a publication ready thesis; i.e., one that needs little additional work by their supervisor.

Interestingly, the data showed that students with less experienced supervisors were more likely to publish than students with experienced supervisors. The higher publication rates for students supervised by ECR staff on the face of it appears counterintuitive. It could be expected that a more experienced supervisor, with a larger publishing track record and established research programs would have more students publishing papers. However, it is possible that ECR academics, who may not have large research teams and research funding may be more reliant on honours students to drive their primary research and are more likely to get publishable data from their projects. They may also provide more mentoring in the student’s preparation of their thesis.

There are a number of limitations with this study, through which the results need to be viewed. Data was collected retrospectively and limited data was available from a single university which may limit the generalisability of study findings. Next, the study used student domestic or international status and the geographic location of a supervisors PhD as a proxy for English language skills. It is possible for a student who undertakes high school study in Australia to have poor written and oral communication skills, and likewise, being an international student does not preclude them from having high levels of English ability. A supervisor who completed their PhD overseas may have done so in an English-speaking country (United Kingdom or USA) or come from a country where English is a common additional language. The study did not take into account the support, or lack of support, students may gain from the entire supervisory team. All students had a co-supervisor as well as their primary supervisor, and the co-supervisors’ characteristics did not inform this study. Likewise, the level of involvement of the co-supervisor in assisting the honours student was not considered, nor any additional support that may have been provided to the student from the supervisor’s group of postdoctoral researchers and PhD students. The study did not examine the impact that individual supervisors may have had on each student’s performance. Regardless of supervisor experience, academic role, or where they completed their PhD, there may be significant differences between individual academic staff members based on their level of effort and time available in supporting their student and their access to resources. Finally, the study did not examine the effect of project feasibility or difficulty. While an honours thesis and presentation can be prepared based on a project that produces few results, had only a small sample size, or resulted in failed laboratory experiments, there may be a specific benefit to those projects that are based on studies that are easy to undertake and generate a lot of useful results. Despite these limitations, our study was comprehensive in capturing all honours students over the study period.

## Conclusion

To our knowledge, this is a first study to explore factors that may affect undergraduate research student performance in a pharmacy degree. The key finding is that all honours students can perform highly and complete honours projects. Factors that may influence student performance include gender, type of honours project, and whether the student had published a peer-reviewed journal article before, during, or after undertaking their honours research. Future curriculum efforts should focus on standardised training opportunities to mitigate the impact of these factors on student performance.

## Data Availability

The data that support the finding of this study are available on request from the corresponding author, N.J.W.
